# Molecular Dynamics Simulation: Comparative Investigation of Nanoscale CuAl_2_ and Fe Interlayers for Interfacial Strengthening–Toughening of Cu/Al Laminated Composites

**DOI:** 10.3390/ma19194126

**Published:** 2026-09-27

**Authors:** Kun Qiao, Haitao Gao, Hailiang Yu

**Affiliations:** State Key Laboratory of Precision Manufacturing for Extreme Service Performance, Light Alloy Research Institute, Central South University, Changsha 410083, China

**Keywords:** Cu/Al laminated composites, molecular dynamics simulation, CuAl_2_ intermetallic compound, Fe interlayer, interfacial strength

## Abstract

The brittle nature of CuAl_2_ intermetallic layers severely limits the service reliability of Cu/Al clad materials in power electronics. Here, molecular dynamics simulations are performed to clarify the thickness-dependent deformation behavior of CuAl_2_ interlayers (0–40 Å) and to propose a ductile Fe interlayer modification scheme to overcome its intrinsic brittleness. Using identical model dimensions, EAM potentials, and loading conditions, the optimized 20 Å CuAl_2_ is set as the unified reference, and gradient Fe interlayers (10–50 Å) are quantitatively compared. The results reveal that CuAl_2_ exerts a non-monotonic regulation: the 20 Å interlayer achieves optimal strength–ductility synergy, while thicker layers (>20 Å) induce brittle cleavage. Substituting the 20 Å CuAl_2_ with an Fe interlayer of equal thickness improves peak tensile strength by 33.5% and suppresses the brittle-damage tendency under the present fully periodic simulation conditions. The 40 Å Fe interlayer exhibits the best comprehensive performance, delivering a 67.2% strength enhancement while maintaining comparable uniform plasticity. Crucially, unlike CuAl_2_ (effective only within 10–20 Å), Fe interlayers sustain favorable synergy across a broad 10–50 Å window. This superior performance originates from triple synergies: physical isolation buffering lattice mismatch, thickness-modulated slip transfer, and a fundamental shift in failure mode from cleavage to matrix-dominated plastic instability. Because periodicity constrains through-thickness crack propagation, this result represents a comparative trend under the specified simulation conditions rather than proof of crack immunity under free-surface conditions. This work provides atomic-scale guidance for interface design in high-performance Cu/Al laminates.

## 1. Introduction

Cu/Al laminated composites combine the excellent electrical and thermal conductivity of copper with the low density and cost advantages of aluminum, holding great promise for high-voltage power transmission, high-density electronic packaging, and interconnects in new-energy power batteries [1,2,3,4]. During rolling and heat treatment of Cu/Al composite sheets, atomic interdiffusion at the interface readily occurs, generating brittle intermetallic compounds (IMCs) such as CuAl_2_, CuAl, and Cu_9_Al_4_. It was long believed that these IMCs, characterized by high hardness and poor fracture toughness, inevitably induce interfacial stress concentration and microcrack initiation; research efforts were consequently directed at suppressing IMC formation and growth as the primary optimization strategy [5,6,7].

With the advancement of in situ characterization techniques, extensive microscale experiments have revealed that the IMC layer thickness exerts a non-monotonic influence on interfacial mechanical properties. When the thickness lies within a critical range of 1–4 μm, thin IMC layers establish robust metallurgical bonding through semi-coherent interfaces and alleviate deformation incompatibility between the two phases, simultaneously boosting interfacial shear strength and plasticity by 20–40%. Only beyond this critical threshold does cleavage fracture, dominated by the brittle phases, lead to a sharp degradation in interfacial performance [8,9,10,11,12]. This finding overturns the conventional wisdom that “the thinner the IMC, the better the performance” and exhibits a pronounced size dependence: as the characteristic length scale shrinks to the nanoscale, the interfacial fraction rises dramatically and deformation behavior becomes governed by size effects and interfacial energy, resulting in a substantially reduced optimal critical IMC thickness [13,14,15]. Among the various Cu–Al intermetallics, CuAl_2_ (θ-phase intermetallic compound) possesses strong thermodynamic stability and a high growth rate, serving as the dominant continuous phase within the IMC layer at conventional low-to-moderate-temperature solid-state composite interfaces. Phases such as CuAl and Cu_9_Al_4_ have higher formation activation energies and narrow formation windows; they precipitate only as discrete minor phases at localized interfacial regions, rarely forming continuous load-bearing layers, and therefore contribute minimally to the overall interfacial mechanical response [16,17,18,19]. The thickness of nanoscale CuAl_2_ interlayers directly governs interfacial strength and brittleness, and elucidating the microscopic mechanisms that regulate their growth represents the core pathway for breaking through the interfacial performance bottleneck of Cu/Al composites [20].

State-of-the-art in situ characterization techniques suffer from limited spatiotemporal resolution, making it difficult to capture the instantaneous dynamic interactions between dislocations and IMC interfaces during deformation. Consequently, the microscopic mechanisms by which CuAl_2_ layers of different thickness regulate dislocation slip, strain localization, and crack propagation remain elusive [21,22]. Molecular dynamics (MD) simulations, offering atomic-scale resolution, provide a powerful means to address this issue [23,24]. Previous MD studies have confirmed that the CuAl_2_ interlayer thickness induces a non-monotonic variation in system strength, and that an appropriate thickness can achieve a synergistic enhancement of strength and toughness through crack-tip blunting. However, most existing work has relied on discrete thickness values to statistically analyze macroscopic properties, without systematically unraveling the coupling among interlayer thickness, microscopic deformation mechanisms, and macroscopic mechanical response. The critical condition for the transition of the interface from coordinated plasticity to brittle failure remains incompletely understood [18,25,26,27,28].

Even when the CuAl_2_ interlayer is tuned to its optimal thickness, the system approaches its intrinsic performance ceiling, offering little room for further improvement. The inherent brittleness of CuAl_2_ cannot be eliminated, and the risk of interfacial cracking under extreme service conditions—such as thermal cycling and impact—remains difficult to fully suppress [5,29]. Developing novel interfacial structures that transcend the performance limits of brittle IMCs is therefore a critical direction in this field. Below 300 °C, metallic Fe exhibits extremely low bulk interdiffusion with both Cu and Al, effectively blocking direct Cu–Al atomic contact and inhibiting the continuous growth of thick, brittle Cu–Al intermetallics such as CuAl_2_. Moreover, BCC-Fe possesses abundant slip systems and exceptional plastic buffering capacity, making it a highly promising candidate for interfacial modification [30,31,32,33]. Nevertheless, existing studies on Fe interlayers typically benchmark against unoptimized, pristine Cu–Al interfaces, rather than adopting the best-performing Cu/CuAl_2_ configuration as a unified reference under identical model dimensions, loading conditions, and interatomic potential frameworks. As a result, the strengthening efficacy and effective tuning range of Fe interlayers have yet to be rigorously verified at the atomic scale [34,35].

To address the two core scientific issues outlined above—(i) the unclear microscopic coupling by which nanoscale CuAl_2_ interlayers regulate interfacial deformation, and (ii) the lack of systematic quantification of the performance advantages of Fe interlayers relative to an optimal CuAl_2_—this study performs two sets of controlled molecular dynamics simulations. First, CuAl_2_ interlayer models with gradient thicknesses spanning 0–40 Å (0–4 nm) are constructed, fully covering the functional regime of nanoscale IMCs from strengthening to degradation. By combining dislocation statistics, shear strain mapping, and defect-evolution analysis, the governing influence of CuAl_2_ thickness on interfacial failure modes is elucidated. On this basis, the 20 Å (2 nm) CuAl_2_ model that delivers the optimal overall performance is selected as a unified benchmark, and Fe interlayer models of gradient thicknesses are constructed under identical matrix dimensions and loading conditions. Both systems are simulated using the same set of embedded-atom method (EAM) potentials, ensuring quantitative cross-comparability. This work aims to quantitatively reveal the interfacial strengthening superiority and optimal thickness range of Fe interlayers at the atomic scale, thereby providing theoretical guidance for the interfacial design of high-performance Cu/Al laminated composite sheets.

## 2. Materials and Methods

Molecular dynamics (MD) simulations were performed to elucidate how interlayer type and nanoscale thickness govern the mechanical properties, deformation behavior, and failure modes of Cu/Al composite interfaces. A Fe interlayer modification strategy is proposed to overcome the intrinsic brittleness limitation of CuAl_2_. A strict single-variable control protocol was implemented throughout model construction to guarantee the comparability of all simulation results.

### 2.1. Atomic Model Construction

All single-crystal laminated models were built using the Atomsk (Beta 0.13.1) software package. Both Cu and Al possess face-centered cubic (FCC) lattices with lattice parameters of 3.615 Å and 4.05 Å, respectively. The lateral dimensions of the simulation box in the X and Y directions were fixed at 100 Å × 300 Å. Along the Z-axis (interface normal), the thicknesses of the Cu and Al matrix layers were held constant at 50 Å, with only the interlayer thickness varied; all other geometric parameters and crystallographic orientations remained unchanged. For the interlayer-free reference, single-crystal Cu and Al were directly bonded at the interface. Introducing an interlayer merely extends the total Z-dimension, thereby strictly satisfying the single-variable constraint. All models were constructed as ideal single crystals without grain boundaries, pre-existing dislocations, or other defects, enabling the intrinsic thickness-dependent deformation mechanisms of the interface to be revealed.

A series of θ-CuAl_2_ interlayer models with gradient thicknesses of 0, 10, 20, 30, and 40 Å was first established. θ-CuAl_2_ adopts a body-centered tetragonal (BCT) structure (a = b = 6.067 Å, c = 4.877 Å), and its thickness was tuned via supercell expansion while maintaining the Cu:Al stoichiometric ratio of 1:2. All interfaces were kept atomically flat and gap-free. This set of models serves to reveal the thickness-dependent evolution of brittle intermetallic compounds (IMCs) and to identify the optimal interlayer dimension for the system. Subsequently, the CuAl_2_ interlayer was replaced by a body-centered cubic (BCC) pure Fe interlayer (lattice parameter: 2.866 Å), and control groups with thicknesses ranging from 10 to 50 Å were constructed at 10 Å intervals. Both model series share a unified crystallographic orientation of [100]//[100], with the interface parallel to the XY plane. The atomic configurations of all models are presented in Figure 1.

### 2.2. Simulation Parameters and Postprocessing

The embedded-atom method (EAM) potential developed by Zhou et al. [36] was uniformly adopted for both the Cu–Al binary system and the Cu–Al–Fe ternary system. Comprehensive benchmark results for this potential are provided in the Supplementary Materials (Tables S1 and S2). This potential can accurately reproduce the lattice parameters, elastic constants, and intrinsic brittleness of Cu–Al intermetallic compounds, and it has been widely used in simulation studies of CuAl_2_ interfaces. For the ternary system, only the interaction parameters related to Fe were supplemented, while the overall framework of the potential remained unchanged. The elastic constants and other fundamental properties of Fe predicted by this potential show good agreement with experimental values, and the potential has been extensively employed in atomistic simulations of Fe-based interfacial deformation. Adopting a unified potential framework eliminates systematic comparison errors that would otherwise originate from the use of different interatomic potentials.

All MD simulations were carried out using LAMMPS (Large-scale Atomic/Molecular Massively Parallel Simulator, 2 August 2023, stable_2Aug2023) with three-dimensional periodic boundary conditions. The fully periodic configuration was chosen to eliminate free-surface effects, ensuring that the observed interfacial deformation originates from the intrinsic lattice mismatch and interlayer properties rather than from artificial boundary relaxation, with a time step of 0.001 ps. The initial structures were first relaxed by energy minimization via the conjugate gradient method to eliminate local residual stresses and unphysical atomic configurations. The systems were then equilibrated at 300 K for 50 ps under an isothermal–isobaric (NPT) ensemble until the total energy and pressure reached stable equilibrium. After full relaxation, uniaxial tensile loading was applied along the Y-axis (parallel to the interface) at a constant strain rate of 1 × 10^9^ s^−1^ up to a total tensile strain of 0.2.

Postprocessing and structural analysis were performed using the OVITO software package (version 3.16.1). The DXA (Dislocation Extraction Algorithm) was used to quantify the evolution of global dislocation density; the atomic shear strain module was adopted to map the local shear strain field; and the PTM (Polyhedral Template Matching) algorithm was applied to identify crystal structures, twin boundaries, and disordered defect regions. All parameters for relaxation, tensile loading, and visualization were kept identical across all models. To eliminate stress baseline deviations caused by differences in total model thickness, the tensile stress of each model was normalized by its cross-sectional area, ensuring the rigor of cross-model comparisons. Unless otherwise stated, the reported results are based on one equilibrated tensile trajectory per thickness and should be interpreted as representative values.

## 3. Results and Discussion

### 3.1. Mechanical Response and Microscopic Deformation Coupling of the Cu/Al–CuAl_2_ Composite System

#### 3.1.1. Macroscopic Mechanical Properties and Dislocation Density Evolution

Three-dimensional fully periodic boundary conditions are deliberately adopted in this work to eliminate the surface size effects that commonly plague nanoscale models with free surfaces. Under this configuration, the deformation behavior of the interface can be captured in its intrinsic state, without artificial stress release or premature damage initiation induced by free surfaces. It is acknowledged that periodic boundaries inherently suppress through-thickness crack propagation; however, the present study focuses on the intrinsic interfacial mechanical response during the uniform plastic deformation stage, where the dominant deformation events—dislocation nucleation, slip transmission, and strain localization—are faithfully represented. This methodological choice ensures that the observed thickness-dependent deformation mechanisms reflect the genuine interfacial behavior rather than being obscured by spurious surface effects. A unified quantitative analysis framework has been established in existing molecular dynamics studies on Cu/CuAl_2_ interlayers: the ultimate strength and uniform plasticity are characterized by the peak stress and peak strain, respectively. Stress oscillations in the high-strain range of 0.1–0.2 merely reflect local reversible defect evolution and are used solely for qualitative auxiliary analysis rather than for quantitative plasticity comparison among different groups [14,18,37]. This identical evaluation standard is also applied to all Fe interlayer models to guarantee fair cross-system comparison. This evaluation criterion is independent of boundary conditions, and its universality has been corroborated by free-boundary fracture models, fully periodic single-crystal and polycrystalline simulations, compression simulations of intermetallic compounds, and molecular fracture theory [38,39,40]. Accordingly, peak stress and peak strain are uniformly adopted in this work to quantify the influence of CuAl_2_ interlayer thickness on strength and plasticity, while post-peak stress fluctuations in the 0.1–0.2 range serve only to assist in elucidating the microscopic mechanism of local damage accumulation in thick IMCs.

Based on the above evaluation criteria, Figure 2a,b present, respectively, the engineering stress–strain curves and the evolution of global dislocation density over the full strain range of 0–0.2 for CuAl_2_ interlayer thicknesses of 0, 10, 20, 30, and 40 Å. The CuAl_2_ interlayer thickness exerts a pronounced non-monotonic influence on the mechanical properties. For the interlayer-free sample (d = 0 Å), the absence of IMC pinning strengthening is offset by the lack of premature local damage induced by the brittle phase, resulting in a peak strength higher than that of the 30 and 40 Å interlayers. The 10 Å thin interlayer achieves the highest peak strength through interfacial pinning. The 20 Å interlayer exhibits a slight decrease in peak strength but yields the largest peak strain among all groups, delivering the optimal synergy of strength and plasticity. When the thickness increases to 30 and 40 Å, the excessive fraction of brittle phase causes a simultaneous deterioration of strength and plasticity, with the 40 Å sample showing the poorest overall performance. Based on these two key indicators, 20 Å is identified as the optimal CuAl_2_ interlayer thickness for the present system.

The dislocation evolution curves also exhibit thickness dependence. In the elastic regime (ε < 0.04), the dislocation density remains generally stable for all cases. For the direct Cu/Al interface (d = 0 Å), a high-density misfit dislocation network forms after relaxation owing to the 10.6% lattice mismatch, with an initial dislocation density of 2.0 × 10^17^ m^−2^. Upon introduction of CuAl_2_, the superposition of misfit strains at the two heterogeneous interfaces raises the total density of native misfit dislocations to 3.2 × 10^17^–3.7 × 10^17^ m^−2^. Once plastic flow initiates, stress and dislocation density increase synchronously, consistent with the Taylor work-hardening law. The initial dislocation density primarily affects the initial defect state, while the multiplication rate of dislocations is mainly controlled by interfacial slip resistance. The interlayer-free model exhibits the fastest dislocation multiplication because of the absence of slip barriers, and a large number of interfacial voids form at ε = 0.08, where damage-induced softening offsets work hardening prematurely. The peak dislocation density of all samples lags behind the peak stress, indicating that microdefect evolution continues into the 0.2 strain range even after the macroscopic load-bearing capacity has been exceeded. In the post-peak stage, the 20 Å interlayer shows a gentle dislocation annihilation rate, corresponding to a higher resistance to the propagation of microvoids and shear bands. The rapid decline in dislocation density for the 30 and 40 Å thick interlayers is consistent with the microscopic observation that thick CuAl_2_ readily accumulates atomic disorder and promotes rapid defect nucleation.

It is worth noting that the optimal interlayer thickness identified here deviates from the characteristic strength thickness of 24.4 Å reported in a previous MD study on the same system [18]; this discrepancy arises primarily from differences in crystallographic configuration, system dimensions, and interatomic potential parameters adopted in the two types of models [41,42]. Different EAM potentials and interfacial lattice configurations alter the interfacial misfit stress and intrinsic brittleness of CuAl_2_, thus shifting the critical optimal thickness for brittle failure.

#### 3.1.2. Atomic-Scale Strain Field Characterization

Macroscopic stress and average dislocation statistics alone cannot spatially resolve the sites of local strain concentration and microdefect nucleation [42]. Integrating the foregoing analysis, ε = 10% encompasses the complete deformation sequence of elasticity, yielding, and peak loading, whereas no new deformation mechanisms emerge in the 0.1–0.2 range, where only reversible damage cycles occur. Accordingly, atomic shear strain maps at four critical strains—ε = 4.0%, 6.5%, 8.5%, and 10.0%—are extracted and displayed in Figure 3. The color gradient from blue to red represents increasing atomic shear strain, and each row corresponds to an interlayer thickness ranging from 0 to 40 Å, fully reproducing the interfacial layered deformation and damage evolution process.

Initial plastic stage (ε = 4.0%): The Cu and Al phases exhibit a lattice mismatch of 10.6%. (a1) For the interlayer-free model, all high-shear-strain regions are concentrated at the direct Cu–Al heterogeneous interface, indicating that this interface is the preferred site for plastic deformation initiation, where local dislocation nucleation occurs first. (a2) The 10 Å interlayer maintains an intact crystal structure, with the load uniformly distributed to the matrix on both sides. (a3)–(a5) Slight plastic deformation emerges within the 20–40 Å interlayers; a thicker interlayer bears a higher proportion of the load, creating a structural predisposition to internal stress concentration at later deformation stages.

Yield hardening stage (ε = 6.5%): (b1) For the d = 0 Å interlayer-free model, slip on the Al side propagates unimpeded to the interface, forming continuous high-strain aggregation zones. (b2) The intact 10 Å interlayer effectively blocks cross-layer slip transmission, with stress concentration confined solely to the thin interfacial layer. (b3) The 20 Å interlayer exhibits a uniform strain distribution across the entire system, where abundant dislocations accommodate the deformation mismatch between the two phases. (b4, b5) For the 30 and 40 Å interlayers, high-strain regions gradually shift into the interior of the brittle interlayer, accompanied by continuously intensifying interlayer deformation mismatch.

Near-peak stage (ε = 8.5%): (c1) For the d = 0 Å model, extensive plastic damage accumulates at the interface, with damage-induced softening surpassing work hardening. (c2) The 10 Å interlayer remains structurally intact without penetrating defects. (c3) Deformation is transmitted smoothly between the Al matrix and the 20 Å interlayer, with barely any shear slip generated on the Cu side. (c4, c5) For the 30 and 40 Å interlayers, shear bands fully penetrate the interlayer, and the damage nucleation core shifts from the interface into the interior of the IMC interlayer.

Initial softening and fracture stage (ε = 10.0%): (d1) For the d = 0 Å model, continuous high-strain damage bands form along the interface, accompanied by large-scale interfacial atomic decohesion. (d2) Local brittle damage regions appear inside the 10 Å interlayer. (d3) Only a small number of high-strain atoms are sparsely distributed in the 20 Å interlayer, giving it the best overall deformation-coordination capacity among all models. (d4, d5) Multiple interconnected shear channels form inside the 30 and 40 Å interlayers, where brittle cleavage fracture becomes the dominant failure mode.

Overall, with increasing CuAl_2_ interlayer thickness, the core of strain concentration gradually shifts from the Cu/Al interface into the interior of the IMC interlayer, and the failure mode transitions from interfacial damage to interlayer cleavage, which is fully consistent with the non-monotonic evolution of macroscopic mechanical properties [14,37].

This shift in strain localization reflects the interplay between interfacial cohesion and defect-mediated deformation. The high work of separation, *Wsep*, for Cu/CuAl_2_ and CuAl_2_/Al listed in Table S2 explains why damage preferentially develops inside thick CuAl_2_ layers, instead of prompt debonding at either heterogeneous interface. Meanwhile, mismatch-induced defect structures and local atomic rearrangement act as sites for slip initiation and strain redistribution. Wu et al. [43] reported a correlation among local stress accumulation, atomic rearrangement, and Shockley partial-dislocation nucleation when depositing Cu-based composite particles onto Al substrates. Even though their impact-driven loading geometry differs from the tensile models used herein, their findings support the mechanistic interpretation of interfacial strain evolution shown in Figure 3. Atomic trajectories reveal local atomic shuffling, whereas no significant long-range interdiffusion occurs at 300 K within the simulated timescale of approximately 250 ps. Taken together, the separation-work data and strain-field analysis link strong initial interfacial cohesion to thickness-governed deformation redistribution, establishing the physical foundation for the three deformation modes described in the following section.

#### 3.1.3. Comprehensive Deformation Mechanism of CuAl_2_ Interlayer Thickness Regulation

Integrating the multiscale characterization of macroscopic mechanics, dislocation evolution, and microscopic strain fields described above, the CuAl_2_ interlayer regulates the system performance through the dynamic competition of three effects: dislocation hardening, interfacial damage softening, and IMC brittle fracture. Three typical deformation modes can thus be identified:

Strength-dominated mode (d = 10 Å): The thin interlayer bears a low fraction of the load, permitting sufficient dislocation multiplication in the Cu and Al matrices. The pronounced interfacial pinning strengthening effect yields the highest ultimate strength among all groups. However, slip tends to accumulate locally at the interface, resulting in a relatively limited plasticity reserve.

Strength–toughness matched mode (d = 20 Å): The intact interlayer uniformly accommodates the deformation difference between the two phases, with dislocations distributed homogeneously throughout the system. This avoids premature dislocation tangling and saturation, sustains the work-hardening effect, and simultaneously optimizes the strength and plasticity of the system.

Brittle fracture mode (d > 20 Å): High-modulus CuAl_2_ exhibits poor plastic flow capacity and strongly impedes slip transmission from the matrix. Penetrating shear channels readily form inside the interlayer, leading to an abrupt stress drop in the late tensile stage and a concurrent deterioration of strength and plasticity.

As the interlayer thickness increases, the failure mode gradually transforms from large-scale atomic decohesion at the Cu/Al interface to cleavage fracture inside the interlayer. Even at the optimal condition of 20 Å, the intrinsic brittleness of CuAl_2_ cannot be eliminated, and the effective regulation window is merely 10–20 Å. To address this performance limitation, the present work adopts the 20 Å CuAl_2_ interlayer as a unified control benchmark, constructs composite structures with gradient-thickness Fe interlayers, and investigates the interfacial optimization mechanism of a ductile interlayer.

### 3.2. Mechanical Response and Microscopic Deformation Mechanism of the Cu/Al–Fe Composite System

#### 3.2.1. Macroscopic Tensile Mechanical Behavior

Figure 4a compares the engineering stress–strain curves of the CuAl_2_ and Fe interlayer systems at an identical thickness of 20 Å, with all simulation conditions kept strictly identical. This 20 Å Fe interlayer is selected as a unified reference sample matching the optimal CuAl_2_ thickness to intuitively reveal the general strengthening advantage of ductile Fe interlayers. Subsequent gradient-thickness simulations covering 10–50 Å Fe interlayers further screen the full performance evolution trend, and comprehensive analysis confirms that the 40 Å Fe interlayer achieves the most balanced overall mechanical properties. The 20 Å Fe interlayer achieves a peak tensile strength of 2.63 GPa, representing a 33.5% enhancement relative to the best-performing 20 Å CuAl_2_ interlayer (peak strength of 1.97 GPa). It also exhibits a higher work-hardening rate during the plastic regime, leading to significantly improved load-bearing capacity. The CuAl_2_ system shows a slightly larger peak strain, but the stress drops abruptly after the peak, characteristic of typical brittle instantaneous failure [14]. In contrast, the Fe interlayer achieves a synergistic improvement in strength and plasticity, with only a slight reduction in uniform plasticity despite the substantial strength increase [44,45].

Further comparison between the optimal configurations of the two systems is conducted: the 40 Å Fe interlayer achieves a peak tensile strength of 3.30 GPa and a peak strain of 0.090. Relative to the optimal 20 Å CuAl_2_ interlayer (1.97 GPa), the 40 Å Fe interlayer realizes a 67.2% strength enhancement, while the uniform plastic deformation capacity remains at a comparable level.

Figure 4b presents the tensile stress–strain responses of Fe interlayers with gradient thicknesses from 10 to 50 Å. The mechanical evolution trends are consistent across all specimens. Within the investigated thickness range of 10–50 Å, the peak tensile strength increases monotonically with increasing thickness. The peak strain, which characterizes uniform plastic deformation capacity, also exhibits a slight concurrent increase over the 10–40 Å interval and only a minor decline when the thickness reaches 50 Å. Notably, over the entire thickness range, no simultaneous severe degradation of strength and plasticity occurs, which contrasts sharply with the behavior observed in thick CuAl_2_ interlayers [43]. These multi-thickness comparisons eliminate any incidental bias that might arise from a single-thickness simulation, confirming that the ductile Fe interlayer can stably achieve favorable strength–plasticity synergy over a broad thickness window of 10–50 Å, thereby offering a substantially wider interfacial structural tuning range than the brittle CuAl_2_ interlayer.

In terms of failure mechanism, the CuAl_2_ system fails predominantly due to brittle cleavage within the interlayer, where cracks nucleate and rapidly propagate, causing a precipitous drop in performance with increasing thickness. In contrast, the Fe interlayer possesses good intrinsic plastic flow capability, and failure transforms into matrix-dominated plastic instability. Increasing the Fe interlayer thickness only mildly affects deformation compatibility without triggering a catastrophic performance decline—this constitutes the most fundamental difference between the two interlayer types. The disparity in macroscopic properties originates from atomic-scale local strain evolution and slip transfer behavior. To ensure complete consistency with the analysis conditions used for the CuAl_2_ system, the same four critical tensile strains are selected to extract atomic shear strain maps for visual comparison, as shown in Figure 5.

#### 3.2.2. Evolution of Multi-Stage Atomic Shear Strain Field

In Figure 5, each row corresponds to an Fe interlayer specimen with thicknesses of 10–50 Å, and each column corresponds to characteristic tensile strains of 4.0%, 6.5%, 8.5%, and 10.0%. The 20 Å CuAl_2_ interlayer is used as a reference throughout, enabling a direct comparison of load transfer, strain distribution, and interfacial stability between the two interlayer types under identical strains.

At ε = 4.0% (elastic loading stage): For all Fe interlayer specimens (a1)–(a5), only weak shear deformation occurs in the Al matrix, while the Fe interlayer remains in a fully elastic state. No extensive high-strain clusters are observed at either the Cu/Fe or Fe/Al interfaces. In contrast, the 20 Å CuAl_2_ interlayer already exhibits pronounced strain concentration at the same strain. Macroscopic rolling-bonding experiments have confirmed that direct Cu–Al contact readily induces stress concentration at the CuAl_2_ phase interface, and introducing an Fe-based ductile interlayer to separate the two phases is an effective strategy to alleviate interfacial mismatch stress [31].

At ε = 6.5% (interlayer deformation differentiation stage): The strain distributions among different Fe interlayer thicknesses exhibit notable differences. For the ultrathin 10 Å interlayer (b1), the high interface fraction leads to strain concentration at both interfaces. For d = 20 Å (b2), inclined penetrating shear bands form in the Al matrix. At 30–40 Å (b3, b4), the long-range penetrating shear bands disappear, and strain uniformity improves. For d = 50 Å (b5), intersecting shear bands in the Al side increase, aggravating the interlayer mechanical mismatch. Compared with the 20 Å CuAl_2_ at the same strain, the Fe interlayer systems show significantly reduced interfacial strain concentration and markedly improved interfacial load-bearing performance.

At ε = 8.5% (near-peak stage): The role of the Fe interlayer in regulating slip transfer becomes increasingly pronounced with varying thickness. For d = 10 Å (c1), insufficient stiffness fails to block slip, resulting in continuous shear bands penetrating all three layers (deformation transfer sequence: Al → Fe → Cu). When the interlayer thickness reaches 20 Å and above, the Fe interlayer, possessing higher elastic stiffness, remains in an elastic load-bearing state; the slip deformations in the Al and Cu matrices are completely blocked and evolve independently. The yielding sequence is: the Al matrix deforms plastically first, followed by slip initiation in the Cu matrix, and finally the Fe interlayer enters the plastic regime. In the 20 and 30 Å cases (c2, c3), a few residual trans-layer slip channels persist, but slip transfer is largely suppressed. At d = 40 Å (c4), the slip activities in Al and Cu are completely separated. At d = 50 Å (c5), the extent of the slip band zone on the Cu side expands. At this stage, the 20 Å CuAl_2_ already shows evident stress concentration, whereas all Fe interlayer specimens exhibit no significant stress concentration within the interlayer region, demonstrating their superior stress buffering capacity [46].

At ε = 10.0% (softening and fracture stage): The differences in failure characteristics among Fe interlayer thicknesses are further amplified. For d = 10 Å (d1), shear bands penetrate the Cu matrix, causing a precipitous drop in load-bearing capacity. For d = 20–30 Å (d2, d3), the number of trans-layer connected shear channels decreases, and deformation compatibility improves. At 40 and 50 Å (d4, d5), the slip systems in Al and Cu evolve independently, and the Cu matrix exhibits only mild lattice distortion in a narrow region adjacent to the Fe interlayer. Among them, the d = 40 Å specimen (d4) displays the best strain uniformity, simultaneously achieving stress buffering and slip blocking. In contrast, for the d = 50 Å specimen (d5), the excessively thick Fe interlayer exacerbates the mechanical property mismatch between the two phases; the shear band zone on the Al side expands considerably, local strain accumulation intensifies, and the plastic deformation coordination capability declines, consistent with the slight decrease in peak strain observed at this thickness in the macroscopic tensile curves [47].

The above strain evolution patterns directly reflect the regulatory effect of Fe interlayer thickness on slip transfer: thin interlayers allow deformation transmission, while thick interlayers block slip. These two mechanisms jointly dictate the macroscopic strength–plasticity trends, in full agreement with the stress–strain curves. Since shear strain maps only reveal the spatial distribution of strain and cannot distinguish the evolution of crystal defects such as dislocations and twins, the polyhedral template matching (PTM) algorithm is employed to further analyze the microscopic crystal configuration and defect evolution, as shown in Figure 6.

#### 3.2.3. Microstructure Evolution and Defect Characteristics

The PTM algorithm [48] is used to resolve the microstructural configurations (Figure 6). In Figure 6, each row corresponds to an Fe interlayer specimen with d = 10, 20, 30, 40, and 50 Å, while each column corresponds to the four characteristic tensile strains. Green denotes the FCC Al/Cu matrix, blue represents BCC-Fe, red indicates HCP deformation twins, and gray corresponds to dislocations, interfacial misfit, and various disordered defects.

Elastic loading stage (ε = 4.0%): For all gradient-thickness specimens (a1)–(a5), only a small number of surface atoms in the Al layer undergo slip and generate scattered dislocations. The Fe interlayers of all thicknesses maintain a continuous and intact BCC crystal framework. At both Cu/Fe and Fe/Al interfaces, only an extremely thin intrinsic atomic misfit layer exists. A direct comparison with the 20 Å CuAl_2_ control at the same strain reveals that the Fe interlayer system possesses significantly fewer interfacial defect atoms, directly confirming that the Fe interlayer isolates Cu and Al from direct contact and mitigates lattice mismatch. This intact crystal structure is also consistent with the uniform strain distribution observed in the elastic stage.

Interlayer deformation differentiation stage (ε = 6.5%): The Al matrix enters a fully yielded state, with intensified intralayer slip motion. A large quantity of gray disordered atoms accumulates at the interfaces, forming dislocation entanglements, and sporadic twins appear. For the ultrathin 10 Å Fe interlayer (b1), extensive gray disordered defect layers build up at both interfaces, severely disrupting the interfacial ordered lattice structure and providing abundant sites for slip defect accumulation and multiplication [49]. In contrast, for Fe interlayer thicknesses of 20–50 Å (b2–b5), the bulk lattice of the interlayer remains continuous and intact, with only scattered defect atoms at the interfaces. The thickness of 10–20 Å represents the critical boundary for the onset of plasticity in the interlayer. In ultrathin interlayers, the high interface fraction causes the interfacial energy to dominate lattice stability, lowering the dislocation nucleation barrier and leading to premature plasticity. This microscopic difference explains the globally concentrated strain observed in specimens with low interlayer thickness.

Near-peak stage (ε = 8.5%): The differences in crystal integrity among the Fe interlayers become fully apparent. For d = 10 Å (c1), the BCC lattice of the Fe interlayer is completely fragmented, accompanied by wavy plastic distortion at both interfaces. For d = 20 Å (c2), continuous defect bands form inside the interlayer, disrupting the lattice continuity. For d = 30 and 50 Å (c3, c5), the Fe interlayer crystals remain largely intact, with no internal plastic slip occurring. For d = 40 Å (c4), the lattice remains intact, with only a small number of atoms on the Cu-side interface undergoing slip to form fine twins. The hierarchical distinction in crystal integrity among the various thicknesses corresponds directly to the two types of deformation transfer pathways described earlier, demonstrating the role of interlayer stiffness in regulating slip transmission.

Softening and fracture stage (ε = 10.0%): Plastic deformation is fully developed, and various defects proliferate extensively. In the d = 10 Å specimen (d1), both the Al and Cu matrices are filled with coarse penetrating twins, and the originally continuous Fe interlayer is almost entirely transformed into a disordered defect phase. As the interlayer thickness gradually increases from 20 Å (d2) to 40 Å (d4), the size and density of matrix twins decrease concurrently. In the d = 50 Å specimen (d5), only a few fine, short, and scattered twins remain, and the interlayer integrity is the highest among all models. With increasing interlayer thickness, the Fe layer exerts an increasingly strong constraint on twin nucleation and growth, leading to more gradual and uniform plastic deformation in the matrix. However, the excessively thick 50 Å interlayer exacerbates the mechanical property mismatch between the metals, intensifying local strain accumulation on the Cu side and reducing plastic compatibility, consistent with the slight decline in peak strain at this thickness in the macroscopic curves.

Throughout the entire simulation period, the Al/Fe and Fe/Cu interfaces maintain perfect metallurgical bonding, without any failure features such as penetrating microcracks, in stark contrast to the failure mode of the CuAl_2_ interlayer, which is prone to internal brittle cracking.

#### 3.2.4. Size Effect and Triple Strengthening–Toughening Mechanism of Fe Interlayer

##### Regulation of Nanoscale Thickness on Yield Transfer Sequence

The effective stiffness of the Fe interlayer is governed by nanoscale interface size effects, giving rise to two distinct plastic yielding pathways. For thicknesses below 20 Å, the high interface fraction results in a lower overall equivalent yield strength, with the load transfer sequence being Al → Fe → Cu, which tends to cause global strain concentration. When the thickness reaches 20 Å and above, the Fe interlayer with enhanced elastic stiffness bears loads elastically and markedly suppresses cross-layer slip transmission, so the plastic deformations within Al and Cu matrices evolve mostly independently. Minor through-thickness slip channels can still form locally under high strain, but they no longer dominate the overall deformation behavior. The yielding sequence becomes: Al yields first, followed by slip initiation in Cu, and finally plastic flow in the Fe layer. This sequence enhances the uniform plasticity of the system and delays necking fracture. The thickness of 20 Å represents the critical transition boundary between these two deformation mechanisms, while 10 Å corresponds to the extreme case of an ultrathin interlayer. Although thick Fe layers hinder cross-layer slip, the increased volume fraction of ductile Fe provides more sites for dislocation multiplication and work hardening, leading to the continuous growth of tensile strength across the 10–50 Å thickness range. The abrupt structural changes involving lattice fragmentation observed in the PTM microstructural maps corroborate this thickness-dependent deformation behavior.

##### Triple Synergistic Strengthening–Toughening Mechanism

The aforementioned thickness-dependent transition in deformation sequence originates from the fundamental alteration of dislocation behavior and interface coupling induced by the Fe interlayer at the nanoscale. The underlying microscopic dynamic mechanisms can be summarized as the following three synergistic effects. These also constitute the core distinction between the ductile Fe interlayer and brittle CuAl_2_—the latter relies solely on interfacial pinning strengthening and cannot accommodate deformation through its own plastic flow.

Physical isolation and plastic buffering: BCC α-Fe possesses 48 geometrically equivalent slip systems [50], far outnumbering the 12 slip systems each in Cu and Al [51], and exhibits outstanding screw dislocation cross-slip capability. The Fe interlayer separates Cu and Al from direct contact, establishing two sets of semi-coherent interfaces and effectively alleviating the initial stress accumulation caused by the lattice mismatch of the original interface. Its ample plastic redundancy can absorb the deformation mismatch energy between the two phases, circumventing the instantaneous brittle fracture failure mode triggered by stress buildup in CuAl_2_.

Trans-layer deformation transmission and stress homogenization: For thin Fe interlayers (thickness ≤ 20 Å), plastic flow within the interlayer opens trans-layer slip channels, buffering the deformation mismatch between Cu and Al. For thick Fe interlayers (thickness > 20 Å), slip transmission across the layers is blocked, and the tensile load is uniformly distributed, continuously promoting interfacial dislocation multiplication and fully exploiting the work-hardening enhancement arising from three-phase coupling.

Fundamental transformation of failure mode: The failure of conventional CuAl_2_ systems occurs via large-scale interfacial atomic decohesion or internal cleavage within the interlayer. In all Fe interlayer specimens, no penetrating microcracks are observed within the simulated tensile strain range throughout the entire tensile process, in stark contrast to the failure mode of the CuAl_2_ interlayer, which is prone to internal brittle cracking. Failure transforms into matrix-dominated global plastic instability, and the interfacial deformation compatibility is fundamentally enhanced.

##### Comparison of Regulation Windows Between the Two Types of Interlayers

Using the 20 Å CuAl_2_ model with the best overall performance as the benchmark, the Fe interlayer achieves stable strength–plasticity synergy over the entire thickness range of 10–50 Å, without any simultaneous catastrophic degradation of strength and plasticity. The accessible interfacial tuning window is thus significantly wider than that of the brittle CuAl_2_ interlayer system, which only exhibits a favorable strengthening effect within the narrow range of 10–20 Å. Among the Fe interlayer thicknesses, the 40 Å interlayer simultaneously fulfills the dual roles of slip blocking and stress buffering, effectively suppressing local strain concentration on the Cu-side interface and yielding the best overall interfacial deformation uniformity. It is identified as the optimal thickness for comprehensive performance within the scope of this study. As mentioned in Section 3.2.1, the 20 Å Fe interlayer merely serves as a comparison benchmark against the CuAl_2_ system rather than the optimal Fe configuration. Quantitatively, the 20 Å Fe interlayer reaches a peak tensile strength of 2.63 GPa and peak strain of 0.083, while the optimized 40 Å Fe interlayer achieves a peak tensile strength of 3.30 GPa and peak strain of 0.090. Relative to the 20 Å Fe sample, the tensile strength of the 40 Å Fe interlayer increases by approximately 25.48%, and the peak strain representing uniform plasticity rises by about 8.43%. Compared with the optimal 20 Å CuAl_2_ configuration, the 40 Å Fe interlayer realizes prominent strengthening without obvious loss of uniform plasticity. When the interlayer thickness is increased to 50 Å, the mechanical property mismatch between the matrix and the thick Fe interlayer is aggravated, leading to noticeably intensified local strain accumulation on the Al side. Macroscopically, this is manifested as a slight decline in the peak strain that characterizes the uniform plastic deformation capacity.

The above-described thickness-dependent mechanical responses are obtained under unified molecular dynamics simulation conditions. Alongside the equilibrium benchmarks in Tables S1 and S2, the equilibration and trajectory analyses provide complementary evidence of the potential’s applicability to the present deformation study. All layered models retained structurally stable initial configurations during 50 ps of NPT equilibration at 300 K. Using a common temperature and loading protocol then enables the interlayer type and thickness to be compared on a consistent basis. Strain rate is also relevant to the interpretation of deformation mechanisms: Malti et al. [52] demonstrated strain-rate-dependent competition between twinning and phase transformation in Ta-based systems. At the present strain rate of 1 × 10^9^ s^−1^, PTM analysis identifies stacking-fault and twin regions in the FCC matrices, while the α-Fe layer retains its BCC structure before localized plastic disorder develops; no bulk phase transformation is detected. The reported thickness-dependent responses are therefore evaluated under this common MD loading condition, with absolute stress levels and kinetic details potentially differing at experimental strain rates.

## 4. Conclusions

In this study, molecular dynamics simulations were employed to systematically elucidate the regulatory effect of nanoscale CuAl_2_ interlayer thickness on the mechanical behavior of ideal single-crystal Cu/Al composite systems free of grain boundaries and pre-existing defects. Adopting the optimal 20 Å CuAl_2_ interlayer as a unified benchmark, the interfacial strengthening efficacy and microscopic mechanisms of Fe interlayers with gradient thicknesses were quantitatively compared under strictly identical simulation conditions. Through multiscale characterization integrating macroscopic stress–strain curves, dislocation statistics, atomic shear strain fields, and PTM-based crystal defect analysis, the core conclusions are drawn as follows:

(1) The nanoscale CuAl_2_ interlayer exerts a non-monotonic regulation on the mechanical properties of Cu/Al composites. An optimal thickness of 20 Å achieves synergistic strength–ductility enhancement through homogeneous strain accommodation; thinner interlayers (<20 Å) strengthen the interface primarily via dislocation pinning, whereas thicker interlayers (>20 Å) induce brittle cleavage within the IMC layer, causing a simultaneous degradation of strength and ductility and a transition in failure mode from interfacial decohesion to intra-layer fracture.

(2) For the single-crystal nanoscale composite structure investigated here, replacing the optimal 20 Å CuAl_2_ interlayer with a 20 Å Fe interlayer raises the peak tensile strength to 2.63 GPa—a 33.5% increase—and suppresses the instantaneous brittle-damage tendency typical of CuAl_2_ under the present fully periodic simulation conditions. Notably, the Fe interlayer sustains a favorable strength–ductility synergy across a wide thickness window of 10–50 Å, substantially exceeding the narrow 10–20 Å effective range of CuAl_2_. The 40 Å Fe configuration delivers the best overall performance, attaining a peak strength of 3.30 GPa and a peak strain of 0.090, which corresponds to a 67.2% strength enhancement over the optimal CuAl_2_ interlayer while retaining comparable uniform plasticity.

(3) The superiority of the Fe interlayer originates from a triple synergistic mechanism: physical isolation buffers the intrinsic lattice-mismatch stress between Cu and Al; thickness-dependent modulation of slip transfer (trans-layer transmission in thin interlayers versus effective blocking in thick ones) homogenizes deformation; and the failure mode fundamentally transforms from brittle cleavage to matrix-dominated plastic instability, thereby effectively mitigating the inherent brittleness bottleneck. These findings provide atomic-scale theoretical guidance for the interfacial design of high-performance Cu/Al laminated composites.

Nevertheless, periodic boundaries constrain through-thickness crack propagation, and the present results should therefore be interpreted as evidence of the relative suppression of brittle damage by Fe within the controlled periodic model, rather than as a universal prediction of fracture resistance under free-surface conditions. Free-surface and seeded-crack simulations are required for further validation.

## Figures and Tables

**Figure 1:**
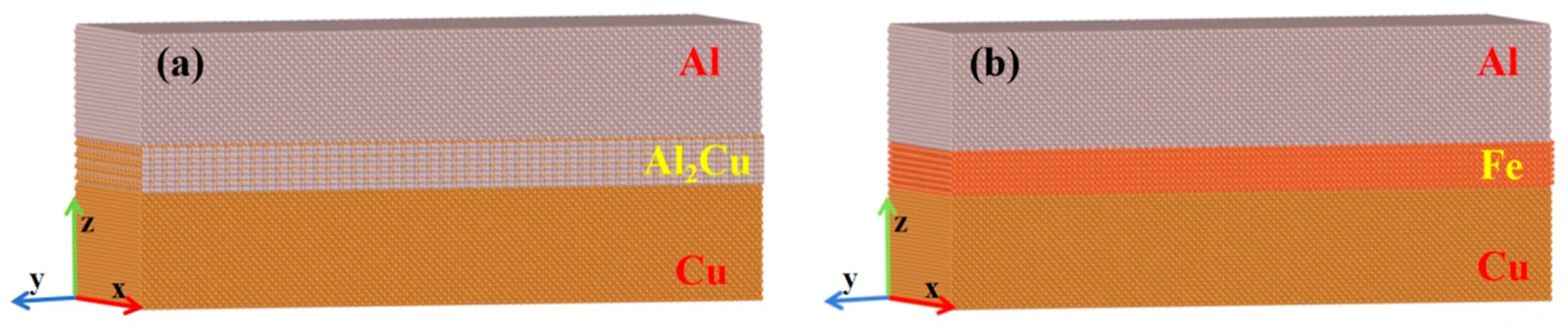
(**a**) Atomic laminated model of the Cu/Al composite with a CuAl_2_ interlayer; (**b**) atomic laminated model of the Cu/Al composite with a Fe interlayer.

**Figure 2:**
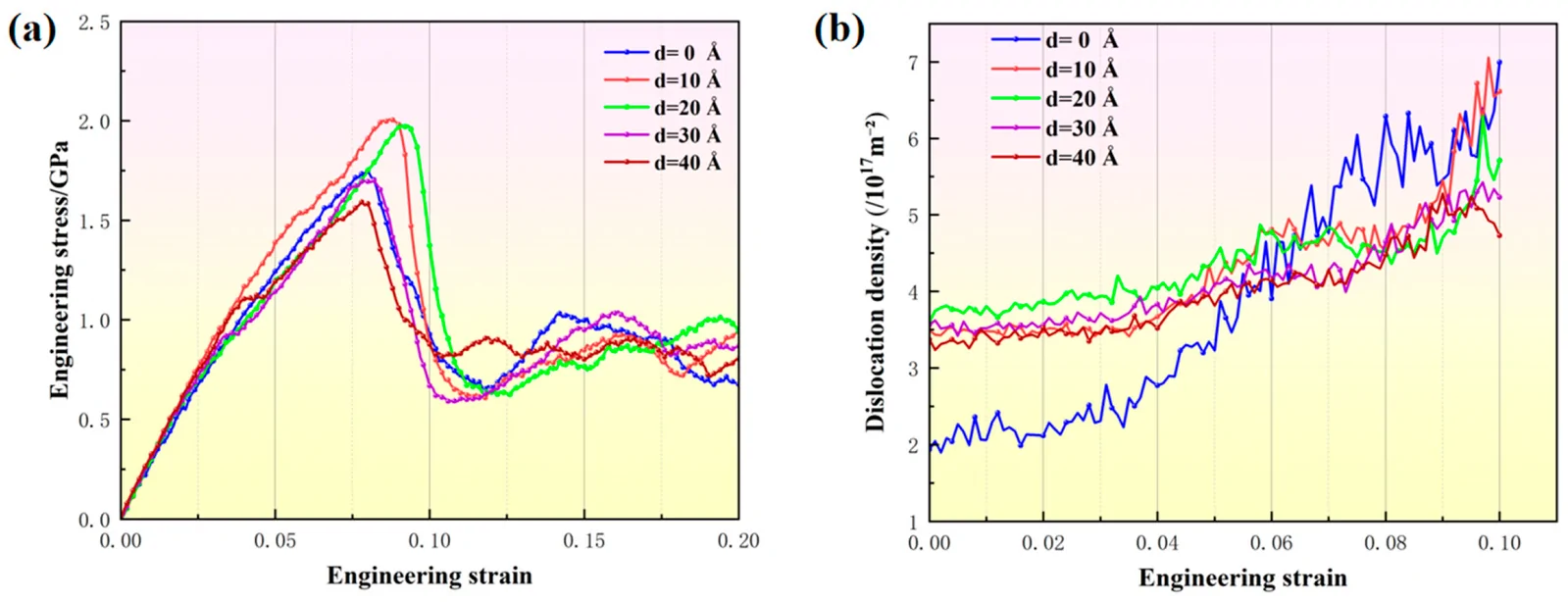
(**a**) Engineering stress–strain curves (full tensile strain range 0–0.2) of Cu/Al composite plates with CuAl_2_ interlayers of various thicknesses; (**b**) corresponding interfacial dislocation density evolution curves recorded over the full 0–0.1 strain interval during tensile deformation.

**Figure 3:**
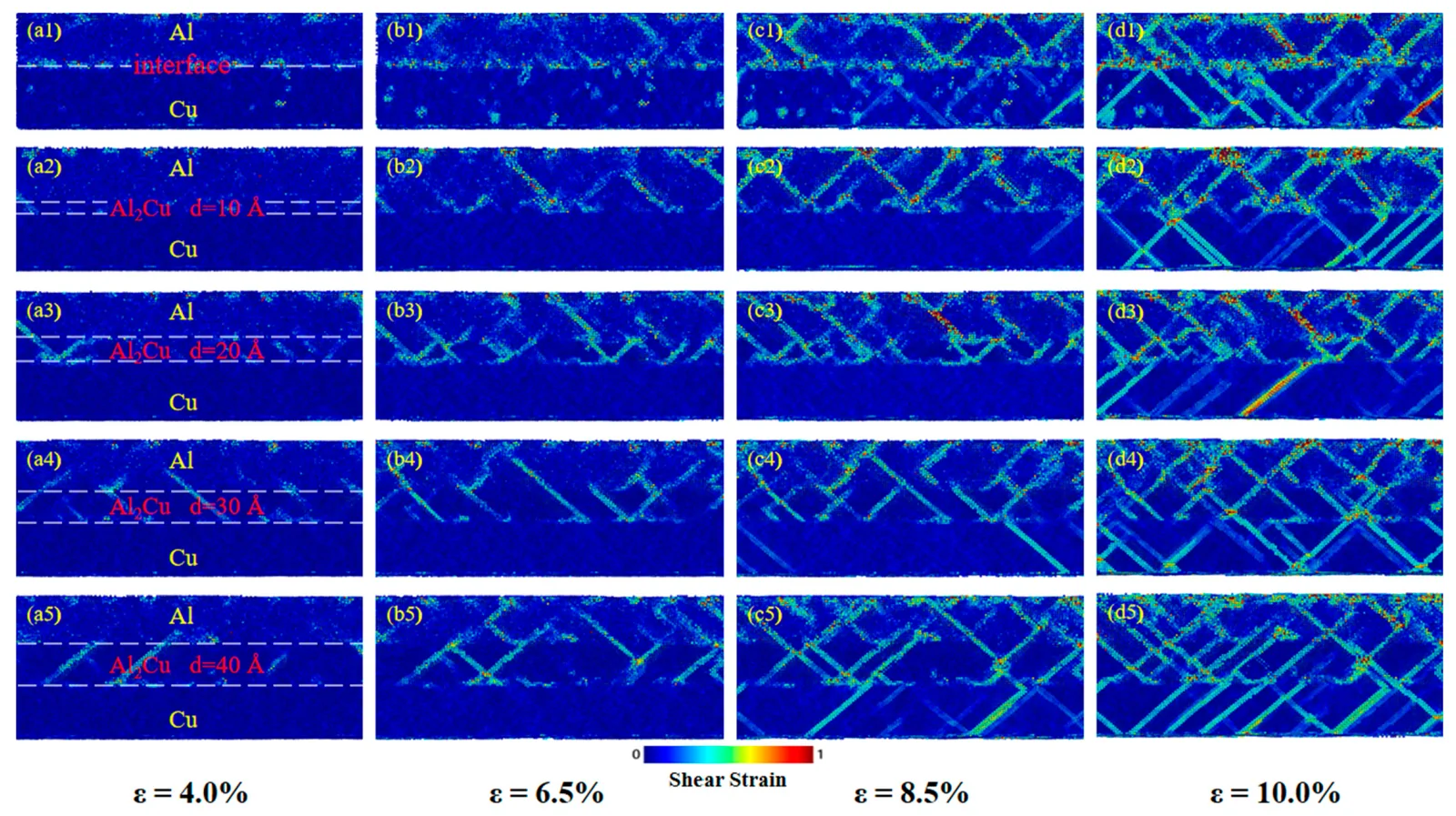
Atomic shear‑strain maps for Cu/Al composites with interlayer‑free, 10 Å, 20 Å, 30 Å and 40 Å CuAl_2_ interlayers under representative tensile strains. (**a1**–**a5**) ε = 4.0%; (**b1**–**b5**) ε = 6.5%; (**c1**–**c5**) ε = 8.5%; (**d1**–**d5**) ε = 10.0%. Each row displays samples following the above‑listed configuration order.

**Figure 4:**
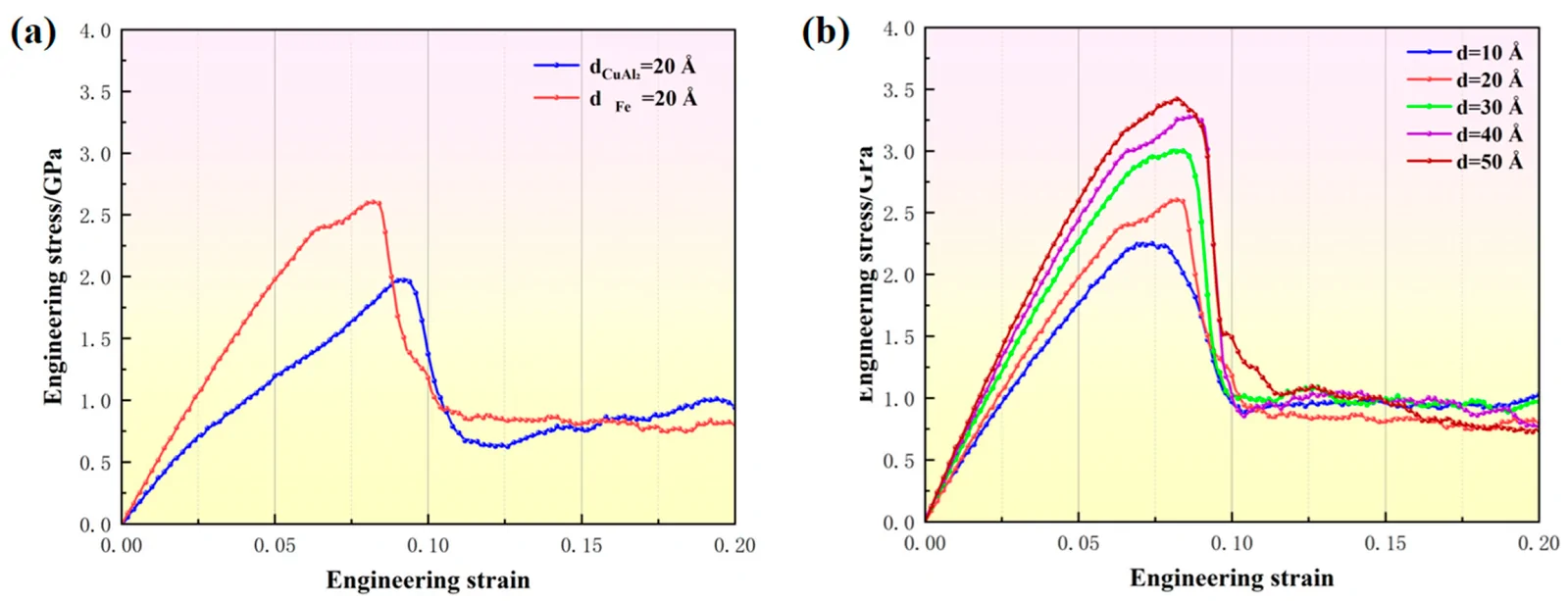
Tensile mechanical responses of Cu/Al composites with different interlayers: (**a**) Stress–strain comparison (strain range 0–0.2) of samples containing 20 Å CuAl_2_ and 20 Å Fe interlayers; (**b**) Stress–strain curves (strain range 0–0.2) of Cu/Al composites with Fe interlayers of various thicknesses.

**Figure 5:**
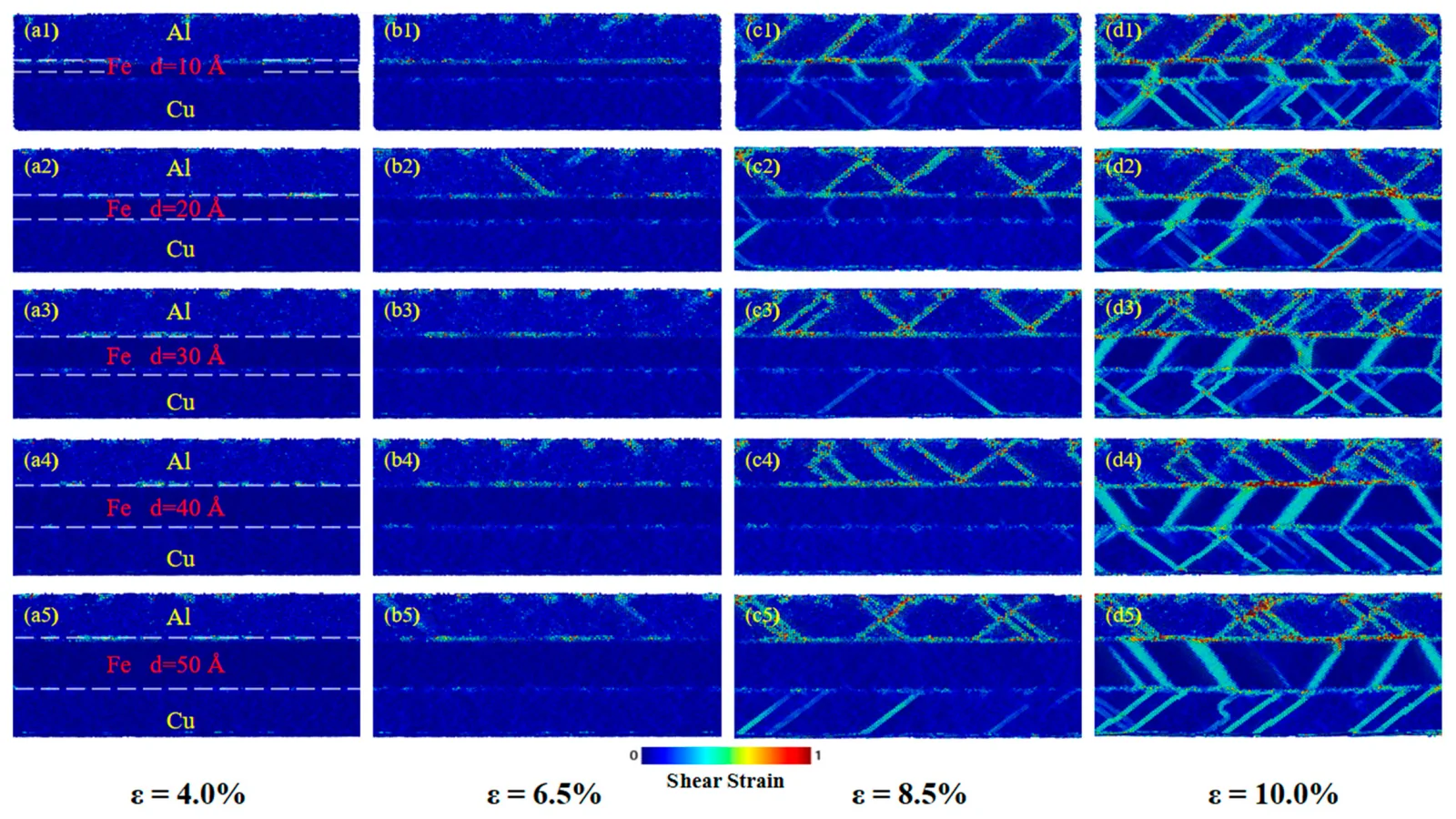
Atomic shear‑strain maps for Cu/Al composites with 10 Å, 20 Å, 30 Å, 40 Å and 50 Å Fe interlayers under representative tensile strains. (**a1**–**a5**) ε = 4.0%; (**b1**–**b5**) ε = 6.5%; (**c1**–**c5**) ε = 8.5%; (**d1**–**d5**) ε = 10.0%. Each row displays samples following the above‑listed thickness order.

**Figure 6:**
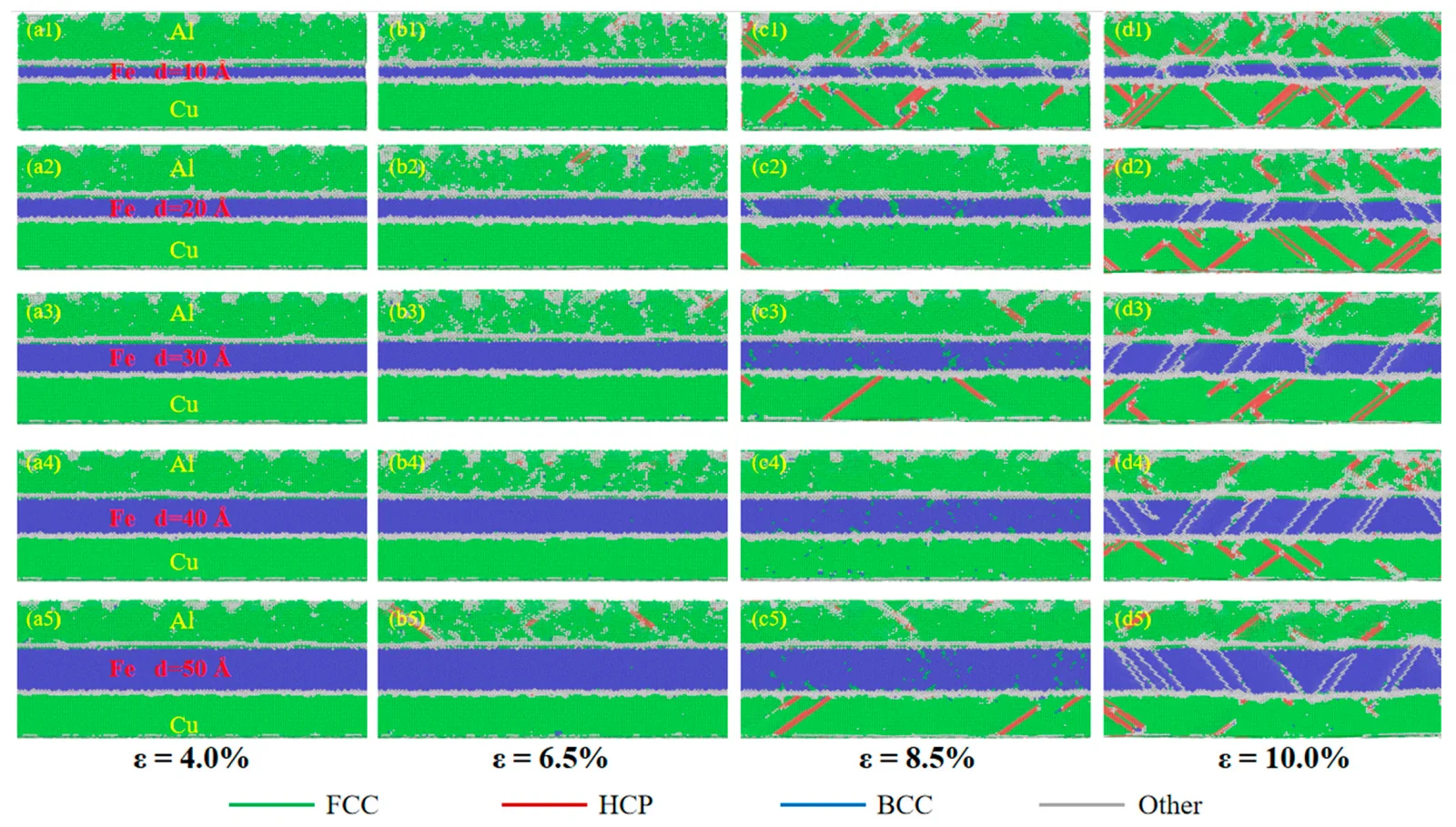
Microstructural configurations obtained by the PTM algorithm for Cu/Al composites with 10 Å, 20 Å, 30 Å, 40 Å and 50 Å Fe interlayers under representative tensile strains. (**a1**–**a5**) ε = 4.0%; (**b1**–**b5**) ε = 6.5%; (**c1**–**c5**) ε = 8.5%; (**d1**–**d5**) ε = 10.0%. Each row displays samples following the above‑listed thickness order.

## Data Availability

The original contributions presented in the study are included in the article/Supplementary Material. Further inquiries can be directed to the corresponding authors.

## Supplementary Materials

The following supporting information can be downloaded at: https://www.mdpi.com/article/10.3390/ma19194126/s1, Table S1. Validation of CuFeAl.eam.alloy potential against reference data for pure-element properties; Table S2. Validation of intermetallic phase and interfacial properties calculated by EAM potential; Table S3. Sensitivity of the calculated work of separation to registry, termination, and relaxation protocol; Table S4. Registry and orientation dependence of the Cu/α-Fe work of separation. References [53,54,55,56,57,58,59,60,61,62,63,64,65,66,67,68,69,70] are cited in the Supplementary Materials.
