# Supplementary material for: Molecular Dynamics Simulation: Comparative Investigation of Nanoscale CuAl2 and Fe Interlayers for Interfacial Strengthening–Toughening of Cu/Al Laminated Composites
Source: Materials (Basel). 2026 Sep 27;19(19):4126. doi: 10.3390/ma19194126 (PMC13665942; doi:10.3390/ma19194126)
Supplement: Supplementary file 1 [file materials-19-04126-s001.zip › materials-4558648-supplementary.pdf]

Supplementary Materials:

Table S1. Validation of CuFeAl.eam.alloy potential against reference data for pure-element properties

Validation category	Parameter	Cu calculated value	Al calculated value	Fe calculated value	Reference value	Deviation	Remarks
Pure-element structure	Lattice structure / constant	fcc; 3.62 Å	fcc; 4.05 Å	bcc; 2.87 Å	Room-temperature values: 3.62 / 4.05 / 2.87 Å [1]	Cu 0.00%; Al 0.00%; Fe 0.00% (given precision)	Good agreement of lattice parameters
Pure-element energy	Cohesive energy (absolute value)	3.54 eV/atom	3.58 eV/atom	4.29 eV/atom	3.49 / 3.39 / 4.28 eV/atom [1]	Cu +1.43%; Al +5.60%; Fe +0.23%	Cu and Fe agree well; moderate deviation for Al
Pure-element EOS	Bulk modulus B	135.20 GPa	72.30 GPa	167.56 GPa	Cu/Al: 137.10 / 76.40 GPa [2]; Fe: 173.10 GPa [3]	Cu −1.36%; Al −5.33%; Fe −3.20%	Compressive properties are close to literature data
Pure-element elasticity	C11/C12/C44	169.63 / 122.14 / 75.74 GPa	105.93 / 60.03 / 27.99 GPa	229.64 / 135.51 / 116.76 GPa	Cu, Al [2]; Fe [3]	Cu +0.45%~+0.73%; Al −1.10%~−1.43%; Fe −1.88%~−5.54%	Elastic constants are consistent with references; note temperature effect
Pure-element elasticity	VRH Young's modulus E	128.22 GPa	69.60 GPa	209.40 GPa	Converted from Ref. [2,3]: 127.35 / 70.38 / 223.41 GPa	Cu +0.68%; Al −1.11%; Fe −6.27%	Stiffness results are reliable
Pure-element elasticity	VRH Poisson's ratio ν	0.35	0.35	0.29	Converted from Ref. [2,3]: 0.35 / 0.35 / 0.28	Cu −0.01%; Al −0.12%; Fe +2.11%	Good agreement for Poisson's ratio

Validation category	Parameter	Cu calculated value	Al calculated value	Fe calculated value	Reference value	Deviation	Remarks
Pure-element defects and stability	Single-vacancy-formation energy, Born criteria	1.28 eV; Passed	0.66 eV; Passed	1.68 eV; Passed	Vacancy-formation energy [4-6]; Born criteria [2,3]	Vacancy-formation energy: Cu −1.77%; Al −0.66%; Fe +19.70%; No numerical deviation for Born criteria; all mechanical-stability conditions are satisfied	Cu and Al vacancy energies match well; large deviation for Fe

Note. Table S1 summarizes the benchmark results of lattice constants, elastic constants and vacancy-formation properties for pure Cu, Al and Fe. All calculations are performed at 0 K after full structural relaxation. Reference data are taken from the literature cited in the main manuscript.

Table S2. Validation of intermetallic phase and interfacial properties calculated by EAM potential

Validation object	Parameter	Calculated result	Reference value	Deviation	Remarks
$CuAl_2$ phase	0 K relaxed lattice constant	a=b=6.16 Å; c=4.98 Å (0 K relaxed)	XRD refinement I4/mcm: a=6.07 Å; c=4.88 Å [7]; DFT-PBE: a≈6.04 Å; c≈4.93 Å [8]	vs. XRD: a +1.57%, c +2.08%; vs. DFT: a +2.02%, c +1.03%	Good agreement with XRD and DFT, supports subsequent simulations
$CuAl_2$ phase	Bulk modulus	EOS-B0 =90.24 GPa; finite-strain B=91.30 GPa	Experimental single-crystal Cij derived BH≈108.60 GPa [8-9]; DFT-BH≈90.50 GPa [8]	−16.00% to −16.90% vs. experiment; −0.30% to +0.90% vs. DFT	Close agreement with DFT; absolute $CuAl_2$ stiffness is underestimated relative to experiment, while all thickness models use the same material description

Validation object	Parameter	Calculated result	Reference value	Deviation	Remarks
$CuAl_2$ phase	Dynamical assessment	Lowest non-translational Γ-point mode: 0.94 THz; minimum signed frequency near M: −0.62 THz; $CuAl_2$ interlayers remain ordered during 50 ps NPT equilibration at 300 K	Positive elastic constants from DFT, satisfying mechanical stability [8-9,15]	Not applicable; qualitative finite-wave-vector assessment	Stable at Γ with localized M-region softness; conclusions are restricted to the interface-constrained models and simulated conditions
$CuAl_2$/metal interface	Work of separation, Wsep	5.03 J/m² (coherent (001)-type configuration, fully relaxed at 0 K)	experimental orientation background [10]; first-principles study on θ-Al_2Cu/Cu interface [11]	Not calculated because the literature interface configurations are not equivalent	Configuration-specific EAM result used for controlled internal comparison
$CuAl_2$/metal interface	Work of separation, Wsep	5.88 J/m² (coherent (001)-type configuration, fully relaxed at 0 K)	DFT studies on coherent Al/Al_2Cu interfaces [12]; interface property depends on stacking-registry and termination	Not calculated because the definition and atomic configuration are not equivalent	Configuration-specific EAM result used for relative interface-strength comparison
Cu–Fe–Al heterogeneous interface	Work of separation, W_{sep}	2.85 J/m² (coherent Fe(001)/Al(001)-type configuration; common	Coherent Fe(001)/Al(001) DFT registries (bcc, bridge, and top): 0.252, 0.145, and 0.093 eV/Å²,	Not calculated because the registry and coherency states are not identical	The EAM value lies within the complete coherent DFT registry range. This is a magnitude validation; the semi-

Validation object	Parameter	Calculated result	Reference value	Deviation	Remarks
		(001) orientation and matched work-of-separation definition)	respectively (4.04, 2.32, and 1.49 J/m²) [13]; semi-coherent Fe/Al DFT: $W_{sep} \approx$ 3.11 J/m² [14]		coherent result is retained only as a qualitative reference.
Cu–Fe–Al heterogeneous interface	Work of separation, W_{sep}	2.02 J/m² (28.80 × 28.80 Å² coherent (001)-type configuration)	DFT Cu(100)/γ-Fe(100): W_{ad} = 3.82 J/m² and literature value 3.89 J/m² [16]; 1.64 J/m² [17]; orientation dependence reported in Ref. [18]	Not calculated because orientations, registries, strain states, and definitions are not fully equivalent	Magnitude is consistent with the DFT energy scale; transferability is restricted to the tested coherent interface family

Note. Table S2 presents the structural, mechanical, and interfacial properties of $CuAl_2$ and representative heterogeneous interfaces. The term "work of separation," Wsep, is used throughout this Supplementary Material to denote the energy required to separate a specified relaxed bonded interface into its corresponding surfaces. The literature values originally defined as work of adhesion, Wad, retain that notation and are compared quantitatively only when the reference states and relaxation definitions are equivalent. Percentage deviations are reported with explicit signs and two decimal places and are omitted for non-equivalent interface configurations or definitions.

Additional Validation and Scope of Applicability

S1. Definitions and Reporting Convention

For each configuration-specific interface comparison, the bonded interface and the corresponding separated slabs contain identical atom numbers and use the same lateral dimensions, coherency strain, interface area, and relaxation protocol. The work of separation, Wsep, is calculated using Eq. (S1):

$$W_{\text{sep}} = \frac{E_{\text{surface},1} + E_{\text{surface},2} - E_{\text{interface}}}{A} \quad \text{(S1)}$$

where Esurface,1 and Esurface,2 are the total energies of the two separated slabs, Einterface is the total energy of the bonded interface system, and A is the interfacial area.

The signed percentage deviations reported in Tables S1 and S2 are evaluated using Eq. (S2):

$$\Delta = \frac{X_{\text{calc}} - X_{\text{ref}}}{X_{\text{ref}}} \times 100\% \quad \text{(S2)}$$

where X_{calc} and X_{ref} denote the calculated and reference values, respectively. All percentage deviations are reported to two decimal places with an explicit sign. A value that rounds to zero is reported as 0.00%, rather than −0.00%. Formal percentage deviations are not reported when the compared configurations, reference states, or property definitions are not equivalent.

S2. Point-Defect, Elastic, Dynamical, and Finite-Temperature Validation

The EAM Fe vacancy-formation energy is 1.68 eV, which is 19.70% higher than the experimental reference value of approximately 1.40 eV. This overestimation predicts fewer thermally generated vacancies and therefore suppresses, rather than exaggerates, vacancy-mediated processes. Neglecting the formation-entropy prefactor, the equilibrium vacancy fraction c_v is estimated using Eq. (S3):

$$c_{\mathrm{V}} \approx \exp\left(-\frac{E_{\mathrm{f}}^{\mathrm{V}}}{k_{\mathrm{B}}T}\right) \tag{S3}$$

where c_v is the equilibrium vacancy fraction, E_f^v is the vacancy-formation energy, k_B is the Boltzmann constant, and T is the absolute temperature. At 300 K, the estimated vacancy fractions are on the order of 10^{-29} and 10^{-24} for formation energies of 1.68 and 1.40 eV, respectively. Both are negligible over the approximately 250 ps simulation duration. The potential should nevertheless not be extrapolated to high-temperature or long-timescale vacancy-controlled behavior without further validation.

No artificial constraint was imposed on Fe migration, and the Cu–Fe and Fe–Al cross-interaction terms were explicitly included in the adopted ternary EAM potential. No significant long-range migration of Fe into the Cu or Al layers was detected at 300 K over the approximately 250 ps simulation window. This observation is specific to the present temperature and timescale and should not be extrapolated to rolling, annealing, or long-term elevated-temperature service.

The $CuAl_2$ bulk moduli obtained independently from the equation of state and finite strain are 90.24 and 91.30 GPa, closely reproducing the DFT value of approximately 90.50 GPa but underestimating the experimental value of approximately 108.60 GPa by 16.00–16.90%. This may affect absolute elastic slopes, stress partitioning, and peak stresses. However, all thickness models use the same potential and crystallographic construction, so the non-monotonic thickness trend is not produced by a changing material description.

Finite-wave-vector phonon calculations for relaxed θ-$CuAl_2$ were performed using a 4 × 4 × 5 conventional-cell supercell containing 960 atoms along the Γ–X–M–Γ–Z path. No non-translational imaginary mode occurs at Γ, where the lowest non-translational frequency is 0.94 THz. Two localized soft branches occur near M, with a minimum signed frequency of −0.62 THz. The interface-constrained $CuAl_2$ layers nevertheless remain ordered during 50 ps of NPT equilibration at 300 K without spontaneous collapse, transformation, or separation. The stability conclusion is therefore restricted to the interface-constrained structures, temperature,

and timescale examined here.

The 0 K equilibrium benchmarks in Tables S1 and S2 are used only to calibrate the potential and are not treated as direct extrapolations to finite-temperature, nonequilibrium deformation. Before loading, every layered model underwent 50 ps of NPT equilibration at 300 K and retained a structurally stable initial configuration without spontaneous phase transformation or premature interfacial separation. At the common strain rate of 1×10^9 s^{-1}, polyhedral template matching identifies stacking-fault and twin regions in the FCC Cu and Al matrices, whereas the α-Fe interlayer retains its BCC structure until localized plastic disorder develops; no bulk phase transformation is detected. Malti et al. [52] demonstrated that strain rate can alter the competition between twinning and phase transformation in metallic systems. Accordingly, the thickness-dependent comparisons made here are valid under the common MD temperature and loading protocol, while the absolute stress level and kinetic details may differ under experimental strain rates.

Unless otherwise stated, one equilibrated production trajectory was analyzed for each interlayer thickness. The initial velocity distribution was not treated as an independent variable, and ensemble-averaged uncertainties were therefore not calculated. The consistent trends across adjacent thicknesses and the agreement among stress–strain, dislocation, atomic-strain, and PTM analyses support the comparative mechanistic conclusions. Nevertheless, the precise defect-nucleation sequence and numerical peak properties may depend on the initial velocity seed; multiple-seed simulations are required for a formal statistical assessment.

S3. Configuration Sensitivity and Dynamic Response of Cu/$CuAl_2$ and $CuAl_2$/Al

A total of 28 bonded-interface configurations were examined across the original large cells and independent termination-test cells. The coherent (001)-type orientation was retained to isolate registry, termination, and relaxation effects without introducing crystallographic orientation as an additional variable. The sensitivity of the Cu/$CuAl_2$ and $CuAl_2$/Al work of separation to registry, termination, and relaxation protocol is summarized in Table S3.

Table S3. Sensitivity of the calculated work of separation to registry, termination, and relaxation protocol.

Interface/test	Configurations	Relaxation	*W*sep result or sensitivity	Main outcome
Cu/$CuAl_2$, original large cell	Four lateral translations	Full relaxation	4.8822–5.0287 J/m^2; −0.88% to +2.09% relative to the unshifted model	The selected x-half-shift model gives 5.03 J/m^2 and is favorable within the tested family.

Interface/test	Configurations	Relaxation	*W*sep result or sensitivity	Main outcome
$CuAl_2$/Al, original large cell	Four lateral translations	Full relaxation	4.0787–5.8810 J/m^2; three registries give 5.4319–5.8810 J/m^2	The selected unshifted model gives 5.88 J/m^2; $CuAl_2$/Al remains stronger in three of four matched comparisons.
Both interfaces, fixed termination	Four translations for each termination	Registry-preserving, normal-only	Variation within any fixed termination ≤0.16%	Lateral registry has a very small effect when termination is fixed.
Both interfaces, Cu- versus Al-terminated $CuAl_2$(001)	Matched termination tests	Registry-preserving, normal-only	Termination changes Wsep by 6.85–12.93%	Termination has a moderate effect, but every tested interface remains substantially bonded.
Cu/$CuAl_2$ and $CuAl_2$/Al termination tests	Independent matched pairs	Full relaxation	Termination differences: 3.26% and 7.98%, respectively	The conclusion regarding strong adhesion is unchanged by the relaxation protocol.

These calculations show that the selected interfaces are energetically favorable and representative within the tested coherent (001)-type family. They are not presented as global minima over all crystallographic orientations.

The static work-of-separation analysis was complemented by inspection of the equilibrated structures and tensile trajectories. The 10.6% lattice mismatch at the direct Cu/Al interface produces an initial misfit-dislocation density of approximately 2.0×10^{17} m^{-2}. After insertion of the $CuAl_2$ interlayer, the two heterogeneous interfaces exhibit initial defect densities of approximately 3.2–3.7 × 10^{17} m^{-2}. These mismatch-derived interfacial structures remain after energy minimization and NPT equilibration at 300 K and participate directly in subsequent slip initiation and strain redistribution.

During tensile loading, the direct Cu/Al interface is the preferred site for defect nucleation and interfacial decohesion. A thin $CuAl_2$ interlayer impedes slip transfer; the 20 Å interlayer promotes the most homogeneous deformation, and thicker interlayers shift strain localization and cleavage into the $CuAl_2$ phase. The high W_{sep} values in Table S2, together with the registry- and termination-sensitivity results in Table S3, explain why thick-interlayer damage develops within $CuAl_2$ rather than through prompt debonding at either heterogeneous interface.

Mismatch-induced defect structures and local atomic rearrangements provide sites for slip

nucleation and strain redistribution. Wu et al. [43] reported a correlation among local stress accumulation, atomic rearrangement, and Shockley partial-dislocation nucleation in impact-loaded Cu-based composite particles on Al. Although that loading geometry differs from the present tensile configuration, it supports the mechanistic interpretation of the interfacial strain fields. The present trajectories capture local atomic shuffling, whereas no significant long-range interdiffusion occurs at 300 K over the approximately 250 ps simulation window. The simulations therefore resolve defect-mediated mechanical evolution under the adopted loading conditions, but do not represent long-timescale, diffusion-controlled interfacial equilibration.

S4. Fe/Al Comparison and Cu/Fe Orientation Sensitivity

For the Fe/Al benchmark, the study of Park et al. [13] provides the closest comparison in both crystallographic configuration and energy definition. Their spin-polarized PBE-DFT calculations used a coherent Fe(001)/Al(001) interface in which fcc Al was represented by a rotated body-centered-tetragonal cell and subjected to approximately 0.88% biaxial compression to match the in-plane periodicity of bcc Fe. Atomic positions were relaxed only along the interface-normal direction, while the in-plane periodicity was fixed. The work of separation was evaluated using matched slab and bonded-interface reference states, consistent with the definition given in Section S1.

Park et al. [13] reported W_{sep} values of 0.252, 0.145, and 0.093 eV/Å^2 for the bcc, bridge, and top registries, respectively, corresponding to 4.04, 2.32, and 1.49 J/m^2. The present EAM result of 2.85 J/m^2 lies within the complete coherent DFT range of 1.49–4.04 J/m^2 and between the bridge and bcc values, supporting a physically reasonable interfacial bonding magnitude.

This comparison is used as a magnitude validation rather than an exact registry-by-registry correspondence because differences may remain in lateral translation, coincidence-cell size, in-plane strain distribution, and surface reference states. Stacking registry modifies local interfacial coordination and bond lengths, whereas semi-coherent interfaces accommodate misfit through dislocations and local relaxation and therefore yield an averaged effective Wsep. The semi-coherent Fe/Al value of 3.11 J/m^2 reported by Czelej and Kurzydłowski [14] is consequently retained only as secondary qualitative support. The present EAM value is configuration-specific to the coherent Fe(001)/Al(001)-type interface used in the tensile simulations. The comparison of these interfacial energy values is summarized in Table S2.

For Cu(001)/α-Fe(001), four in-plane registries were tested in a common coherent supercell of approximately 28.8 × 28.8 Å^2. Atom count, interface area, vacuum thickness, potential, and relaxation procedure were held fixed, and only interface-normal relaxation was allowed to preserve each registry. The resulting W_{sep} values span only 1.950249–1.950778 J/m^2, corresponding to a maximum registry-induced fluctuation of 0.03%. The selected configuration is therefore not an anomalously optimized high-adhesion state. The registry- and orientation-

sensitivity results for the Cu/α-Fe interface are summarized in Table S4.

Table S4. Registry and orientation dependence of the Cu/α-Fe work of separation.

Interface/test	Sampling/model	Relaxation	*W*sep (J/m²)	Relative result
Cu(001)/α-Fe(001) registry scan	Four translations; ≈28.8 × 28.8 Å² cell	Registry-preserving, normal-only	1.950249–1.950778	Maximum registry variation: 0.03%.
Cu(110)/α-Fe(110) registry scan	Six translations; 8 × 4 Cu/5 × 5 Fe match	Registry-preserving, normal-only	1.936777–1.936836	A similarly flat registry dependence is obtained.
(001) versus (110)	Matched low-index orientations	Full three-dimensional relaxation	2.022614 versus 2.008094	(001) is stronger by 0.72%.
(001) versus (110)	Selected registry for each orientation	Registry-preserving, normal-only	1.950249 versus 1.936836	(001) is stronger by 0.69%; the ordering is reproduced independently.

A low-strain Cu(110)/α-Fe(110) model was constructed using an 8 × 4 Cu / 5 × 5 Fe matching scheme, giving coherent strains of −0.45% in Cu and +0.45% in Fe. Under full three-dimensional relaxation, W_{sep} is 2.022614 J/m² for Cu(001)/α-Fe(001), and 2.008094 J/m² for Cu(110)/α-Fe(110), so the (001) interface is stronger by 0.72%. Under normal-only relaxation, the corresponding values are 1.950249 and 1.936836 J/m², preserving a 0.69% difference. Six Cu(110)/α-Fe(110) registries also show negligible variation (1.936777–1.936836 J/m²).

The reproducible ordering $W_{sep}(001) > W_{sep}(110)$ under two independent relaxation protocols, together with the weak registry dependence of both orientations, demonstrates that the relative orientation trend is robust within the tested coherent interface family. The difference between the fully relaxed value (approximately 2.02 J/m²) and the normal-restricted value (approximately 1.95 J/m²) arises from the relaxation constraint rather than registry variation.

All Fe-thickness-gradient models retain the same interface orientation, stacking registry, in-plane dimensions, potential parameters, and relaxation procedure. The systematic changes in interfacial strength, slip transfer, and strain localization are therefore attributed primarily to Fe thickness rather than to uncontrolled structural variation. This validation is restricted to the low-strain coherent Cu/Fe interfaces examined here and does not establish a universal ordering for arbitrary terminations, strain states, or crystallographic orientations; a global transferability benchmark lies outside the scope of the present thickness-dependent deformation study.

Cross-Reference to the Revised Manuscript

Finite-temperature transferability and strain-rate limitations are discussed in Section

3.2.4.3 of the revised manuscript, with Malti et al. cited as Ref. [52]. Dynamic atomic rearrangement, mismatch-induced stress localization, Shockley partial-dislocation nucleation, and the absence of significant long-range interdiffusion over the simulated timescale are discussed in Section 3.1.2, with Wu et al. cited as Ref. [43]. The periodic-boundary-condition limitation and the scope of the Fe-related failure-mode conclusion have been clarified in the Abstract, Section 3.1.1, and the Conclusions. The single-trajectory sampling protocol and its reproducibility limitation are stated in Section 2.2. The newly added passages in the revised manuscript and all additions and revisions in the Supplementary Materials for this round are shown in blue for ease of review.

References

1. Kittel, C. Introduction to Solid State Physics, 8th ed.; Wiley: Hoboken, NJ, USA, 2005; ISBN 978-0-471-41526-8.
2. Simmons, G.; Wang, H. Single Crystal Elastic Constants and Calculated Aggregate Properties: A Handbook, 2nd ed.; MIT Press: Cambridge, MA, USA, 1971.
3. Rayne, J.A.; Chandrasekhar, B.S. Elastic Constants of Iron from 4.2 to 300 K. Phys. Rev. 1961, 122, 1714–1716. https://doi.org/10.1103/PhysRev.122.1714.
4. Berger, A.S.; Ockers, S.T.; Siegel, R.W. Measurement of the monovacancy formation enthalpy in copper. J. Phys. F: Met. Phys. 1979, 9, 1023–1033. https://doi.org/10.1088/0305-4608/9/6/009.
5. Fluss, M.J.; Smedskjaer, L.C.; Chason, M.K.; Legnini, D.G.; Siegel, R.W. Measurements of the vacancy formation enthalpy in aluminum using positron annihilation spectroscopy. Phys. Rev. B 1978, 17, 3444–3455. https://doi.org/10.1103/PhysRevB.17.3444.
6. Kim, S.M.; Buyers, W.J.L. Vacancy formation energy in iron by positron annihilation. J. Phys. F: Met. Phys. 1978, 8, L103–L108. https://doi.org/10.1088/0305-4608/8/5/001.
7. Meetsma, A.; de Boer, J.L.; van Smaalen, S. Refinement of the crystal structure of tetragonal Al_2Cu. J. Solid State Chem. 1989, 83, 370–372. https://doi.org/10.1016/0022-4596(89)90188-6.
8. Fang, X.; et al. Theoretical Prediction of Structural, Mechanical, and Thermophysical Properties of the Precipitates in 2xxx Series Aluminum Alloy. Metals 2022, 12, 2178. https://doi.org/10.3390/met12122178.
9. Eshelman, F.R.; Smith, J.F. Single-crystal elastic constants of Al_2Cu. J. Appl. Phys. 1978, 49, 3284–3288. https://doi.org/10.1063/1.325278.
10. Wang, K.-K. The orientation relationships and interfaces of theta-Al_2Cu/Cu and gamma2-Al_4Cu_9/Cu and the formation sequence at the Cu/Al interface. Thin Solid Films 2021, 717, 138436. https://doi.org/10.1016/j.tsf.2020.138436.
11. Zhang, J.; Wang, A.; Liang, T.; Xie, J.; Zhang, Y. The prediction of Al_2Cu/Cu interfacial structure and properties: A first-principles study. Physica B: Condens. Matter 2023, 661, 414931. https://doi.org/10.1016/j.physb.2023.414931.
12. Huang, Y.; Li, J.; Chen, C.; Zhang, L. Interface properties and nucleation thermodynamics of Al_2Cu precipitated phase by first-principles calculations. J. Mol. Model. 2025, 31, 301. https://doi.org/10.1007/s00894-025-06534-1.
13. Park, S.-D.; Kim, S.Y.; Kim, D. Ab initio investigations of the interfacial bond of Fe(001)/Al(001). Mater. Today Commun. 2021, 26, 102107. https://doi.org/10.1016/j.mtcomm.2021.102107.
14. Czelej, K.; Kurzydłowski, K.J. Ab initio prediction of strong interfacial bonding in the Fe|Al bimetallic composite system. Scr. Mater. 2020, 177, 162–165. https://doi.org/10.1016/j.scriptamat.2019.10.030.

15. Zhang, J.; Huang, Y.; Mao, C.; Peng, P. Structural, elastic and electronic properties of θ (Al_2Cu) and S (Al_2CuMg) strengthening precipitates in Al–Cu–Mg series alloys: First-principles calculations. Solid State Commun. 2012, 152, 2100–2104. https://doi.org/10.1016/j.ssc.2012.09.003.
16. Wang, Y.; Li, M.; Gao, H.; Wang, J.; Sun, B. First-Principles Study on the Cu/Fe Interface Properties of Ternary Cu-Fe-X Alloys. Materials 2020, 13, 3112. https://doi.org/10.3390/ma13143112.
17. Wang, M.-J.; Zhang, G.-W.; Xu, H.; Dong, Y.; Sun, F.-E.; Fu, Y.-Z. Density Functional Theory Investigation on the Stability, Adhesion Strength, Tensile Properties and Fracture Behavior of γ-Fe/Cu Heterogeneous Nucleation Interface. Results Phys. 2021, 24, 104143. https://doi.org/10.1016/j.rinp.2021.104143.
18. Jia, Z.; Zhao, X.; Zhang, G.; Kang, Y.; Xu, H.; Zhao, Z. A comparable study of Fe/Cu interfaces by first-principles method: The surface energy, work of adhesion and electronic structures. Physica B: Condens. Matter 2022, 646, 414348. https://doi.org/10.1016/j.physb.2022.414348.
